# A Pectin-Based Active Coating for Preservation of Fresh-Cut Apples: Incorporated with Luteolin and ε-Polylysine for Enhanced Performance

**DOI:** 10.3390/foods15010063

**Published:** 2025-12-25

**Authors:** Chengheng Li, Junkun Pan, Muhammad Nawaz, Hui Liu, Zhenzhen Lv, Wenbo Yang, Qiang Zhang, Jiechao Liu, Zhonggao Jiao

**Affiliations:** 1 Zhengzhou Fruit Research Institute, Chinese Academy of Agricultural Sciences, Zhengzhou 450009, China; 82101235559@caas.cn (C.L.); pjkshihanhan@163.com (J.P.); mnawazafzal@outlook.com (M.N.); liuhui@caas.cn (H.L.); lvzhenzhen@caas.cn (Z.L.); yangwenbo@caas.cn (W.Y.); zhangqiang02@caas.cn (Q.Z.); jiaozhonggao@caas.cn (Z.J.); 2 Zhongyuan Research Center, Chinese Academy of Agricultural Science, Xinxiang 453000, China

**Keywords:** pectin, edible coating, film, ε-polylysine, luteolin, phenolic compound, fresh-cut apple, anti-browning

## Abstract

Functionalized edible coatings represent a promising strategy to mitigate postharvest losses in fresh and fresh-cut fruits. This study developed a novel, ternary active coating by integrating pectin with a cationic antimicrobial polypeptide (ε-polylysine) and a hydrophobic plant flavonoid (luteolin). The resulting composite film demonstrated transformative improvements in hydrophobicity, antioxidant, and antimicrobial activities as compared with conventional pectin-based films. Specially, the ternary composite film exhibited enhanced barrier performance, reducing water vapor, oxygen and carbon dioxide permeability by 49.1%, 68.6%, and 26.5%, respectively. When applied to fresh-cut apples, the coating effectively suppressed the browning and microbial proliferation while maintaining the hardness, total phenols and flavonoids, total soluble solids, and titratable acids over a 12-day refrigerated storage period. Comprehensive characterization via Fourier transform infrared spectroscopy (FTIR), X-ray diffraction (XRD), scanning electron microscopy (SEM), and molecular docking simulations revealed that these superior functionalities originate from synergistic electrostatic interactions and hydrogen-bonding networks within the ternary matrix. This work provides a practical strategy for designing high-performance, plant-based coatings to reduce food waste and improve the quality of fresh-cut produce.

## 1. Introduction

Fresh-cut apples have become immensely popular in recent years owing to their convenience for consumption, high freshness and nutritive value, and potential health benefits [1,2]. However, they may undergo rapid quality deterioration during storage due to browning and oxidation, softening, microbial spoilage, loss of weight, nutritional value, and flavor. To manage these quality changes and extend the shelf life, many preservation strategies, including blanching, modified-atmosphere packaging (MAP), ultraviolet (UV) radiation, pulsed electric field (PFE), pulsed light (PL), cold plasma, high-pressure carbon dioxide (HPCD), ultrasound, edible coating, and chemical treatment, have been developed [1]. Among these techniques, edible coatings are regarded as one of the most efficient approaches for maintaining the quality of fresh-cut apples [3]. Edible coatings can create a thin layer on the fruit’s surface to form a semipermeable barrier between the fruit and environment, thereby limiting the exposure to oxygen, suppressing respiration rate and metabolic activities, reducing moisture loss, and preventing microbial spoilage and enzymatic browning during storage [4,5]. Commonly, edible coatings are prepared by using edible biopolymers such as polysaccharide and protein either alone or in combination to enhance permeability and mechanical properties to meet the requirements for preserving fresh-cut fruits [5]. Edible coatings also can be functionalized with bioactive compounds, such as antioxidants, essential oils, and antimicrobial agents, to enhance their performance [6,7,8].

Pectin, a naturally occurring heteropolysaccharide primarily derived from fruit peel or pomace, is widely recognized as a promising coating matrix due to its excellent film-forming ability, biocompatibility, biodegradability, and edibility [9,10]. However, single-pectin films and coatings exhibit high hydrophilicity, resulting in poor water vapor barrier property, along with insufficient mechanical strength and a lack of antimicrobial and antioxidant activity, making it challenging to meet the complex preservation requirements of fresh-cut fruits [10,11,12]. To address these limitations, pectin coatings and films are usually functionalized with natural bioactive substances such as antimicrobials and antioxidants [13,14,15,16].

ε-Polylysine is a homo-polymerized polypeptide with broad spectrum of antimicrobial properties. It has been widely employed in the functionalization of polysaccharide-based films and edible coatings, where it serves a dual role as both a cross-linking agent and an antimicrobial agent, thereby enhancing both the barrier and antimicrobial properties [17]. Chang et al. demonstrated that pectin and ε-polylysine could form complexes through electrostatic interactions [18], and the pectin–ε-polylysine complex achieved a better antimicrobial performance than ε-polylysine alone [19]. These findings suggest that ε-polylysine could be a promising antimicrobial ingredient for functionalization of pectin-based edible coatings. However, how ε-polylysine influences the performance of pectin-based coatings and the underlying interaction mechanisms remains to be elucidated.

Phenolic compounds are a group of the most widely distributed bioactives in nature, with notable antioxidant and antimicrobial activities. Previous research demonstrated that they could be incorporated into polysaccharide-based edible coatings and films to modify their physicochemical and functional properties, thereby enhancing disease resistance and antioxidant activity, suppressing browning reactions, and reducing oxidative degradation [20]. However, the effects of incorporating phenolic compounds on polysaccharide-based edible coatings and films are diverse depending on the coating/film matrix and phenolic compounds themselves. This makes it essential to select effective phenolic compounds for incorporating into pectin-based edible coatings for preservation of fresh-cut apples. Therefore, with the aim to identify an effective phenolic compound for incorporation with pectin-based coatings, seven phenolic compounds representing different subclasses, including flavonoids (naringenin, luteolin, and rutin), phenolic acids (protocatechuic, gallic, and chlorogenic acid), and pterostilbene, were selected based on their structural diversity and documented bioactivities relevant to food preservation [21,22].

With the aim of developing a novel pectin-based coating with enhanced performance for the preservation of fresh-cut apples, seven selected phenolic compounds were evaluated for the influence on mechanical and antimicrobial properties of apple pectin–ε-polylysine edible coatings, and then the physicochemical, structural, and functional properties of the screened polyphenol-functionalized pectin–ε-polylysine edible coating as well as its effects on quality preservation of fresh-cut apples were characterized. The findings would provide valuable insights into the functionalization of pectin-based edible coatings with multi-functionality and an applicable approach for the preservation of fresh-cut apples, as well as other fruits.

## 2. Materials and Methods

### 2.1. Chemicals, Reagents and Strains

Apple pectin (AP), gallic acid, pterostilbene, rutin, protocatechuic acid, naringenin, luteolin, and chlorogenic acid were supplied by Shanghai Yuanye Bio-Technology Co., Ltd. (Shanghai, China). ε-polylysine (PL) and luteolin were provided by Shanghai Macklin Biochemical Co., Ltd. (Shanghai, China).

The fungal strain *Penicillium expansum* (ACCC 30904) was bought from the Agricultural Culture Collection of China (ACCC, Beijing, China). *Saccharomyces cerevisiae* and *Aspergillus niger* were obtained from the Fruit Processing Lab, Zhengzhou Fruit Research Institute, CAAS (Zhengzhou, China).

### 2.2. Preparation of Ternary Composite Films

Films were prepared following the method described by Shivangi et al. [23] with slight modifications. Firstly, 4% (*w*/*w*) AP and 4% (*w*/*w*) PL were separately dissolved in distilled water and the pH was adjusted to 8.0 using sodium hydroxide solution. The AP and PL solutions were mixed at the ratio of 3:2 (*v*/*v*). Then, 40% glycerol (on the basis of total mass of pectin and ε-polylysine, *w*/*w*) was added and mixed thoroughly. After that, different concentrations (0.01%, 0.05%, 0.1%, 0.15%, and 0.2%) of phenolic compounds were added and stirred for 60 min. Air bubbles were removed from the film solutions by ultra-sonification for 30 min. Then, 30 g film solution was poured into a 10 cm × 10 cm glass plate. The plate was dried in an oven at 45 °C for 6 h. The dried film was placed in a desiccator at 50% relative humidity (RH) for stabilization for at least 48 h before determination.

### 2.3. Film Performance

#### 2.3.1. Mechanical Properties

The tensile strength (TS), elongation at break (EAB) and thickness of the film (rectangle, 20 mm × 80 mm) were measured using a texture analyzer (TA-XT plus, Stable Micro Systems Ltd., Godalming, UK) following a modified version of the method described by Sun et al. [11]. The initial gauge length was set to 50 mm and the test speed was 50 mm/min. The TS and EAB were calculated using the following equations (Equations (1) and (2)):

(1) $$\mathrm{TS}\;(\mathrm{MPa})\;=\;\frac{F}{a}\times 10^{-6}$$

(2) $$\mathrm{EAB}\;(\%)=\frac{L-L_{0}}{L_{0}}\times 100$$

where *F* is the maximum tensile force at break of film (N); *a* is the cross-sectional area of the film (m^2^); *L* is the length at which the film breaks (mm); and *L*_0_ is the initial gauge length (mm).

The thickness of the film was measured by randomly selecting five positions with an accuracy of 0.001 mm.

#### 2.3.2. Comprehensive Evaluation on the Overall Mechanical Performance of Films

The Simple Additive Weighting (SAW) method [24] was employed to comprehensively evaluate the overall performance of the samples. The procedure consisted of two main steps: In the first stage, since both TS and EAB are beneficial criteria (higher values are desirable), the raw data for TS and EAB were normalized to a 0–1 scale using Equation (3).

(3) R = (X − X_min_)/(X_max_ − X_min_)

where *R* is the normalized value of a sample for criterion, *X* is the original value, and *X_min_* and *X_max_* are the minimum and maximum values of criterion across all samples, respectively.

In the second stage, the overall performance score for each sample was calculated by summing the normalized values of TS and EAB. Both TS and EAB were considered equally important in this study and equal weights were applied. Samples were then ranked based on their overall scores in descending order, with a higher score indicating a more comprehensive performance.

#### 2.3.3. Antimicrobial Activity

The antimicrobial activity of the films was determined against *A. niger*, *P. expansum*, and *S. cerevisiae* according to the method reported by Sharma and Tripathi [25] with minor modifications. Briefly, 2 mL of film solution was added to 50 mL of sterilized, cool melted Sabouraud Dextrose Agar medium. The medium was mixed well, and 15 mL of it was poured into each Petri dish (90 mm in diameter). The media plates were inoculated with 10 mm of fungal mycelial (5-day-old) agar cake. The inoculated plates were incubated at 25 ± 1 °C for 5 days, and then the colony diameter of fungi was measured with a vernier caliper. To evaluate the antimicrobial properties against *S. cerevisiae*, Potato Dextrose Agar medium plates were evenly spread with 150 μL of a yeast suspension (10^6^ cells/mL) and incubated at 25 ± 1 °C for 24 h.

#### 2.3.4. Optical Properties of Films

The color parameters(L*, a* and b*) of the film were determined using a calibrated colorimeter (CS-10, CHN Spec, Hangzhou, China) according to the method used by Bizymis et al. [8]. The color difference (ΔE) was then calculated using Equation (4).

(4) $$\Delta E=\sqrt{{\left(L^{*}-L\right)}^{2}+{\left(a^{*}-a\right)}^{2}+{\left(b^{*}-b\right)}^{2}}$$

where L*, a*, and b* are the color values of the film sample; *L*, *a*, *b* are the values of the standard white board.

The light transmittance and opacity of the films (20 mm × 10 mm) were measured using an ultraviolet-visible (UV-VIS) spectrophotometer in the wavelengths range of 200–600 nm. The opacity of the film was calculated by dividing the absorbance value at 600 nm by the film thickness (mm).

#### 2.3.5. Moisture Content (MC) and Water Solubility (WS)

The MC of the film was determined using the method of Lingait & Kumar [26]. The edible film was cut into circular pieces (d = 2 cm). Each piece was weighed to obtain the initial weight (W_1_) and then dried at 105 ± 1 °C until a constant weight (W_2_) was achieved. The moisture content (MC) was calculated using Equation (5).

(5) $$\mathrm{MC}\;(\%)=\frac{W_{1}-W_{2}}{W_{1}}\times 100$$

The WS was determined using the method of Monteiro et al. [27] with slight modifications. Firstly, a 0.5 g film (W1) was placed in a Petri dish containing 30 mL of distilled water. The dish was then placed at 25 °C and shaken for 24 h. The residual film was obtained by filtering with Whatman filter paper (No. 1) and then dried at 105 ± 1 °C until a constant weight (W2) was achieved. WS was calculated using Equation (6).

(6) $$\mathrm{WS}(\%)=\frac{W_{1}-W_{2}}{W_{1}}\times 100$$

#### 2.3.6. Water Contact Angle (WCA)

The WCA of the films was measured using a contact angle analyzer (OCA 50, Data Physics, Filderstadt, Germany) at 25 °C according to the method of Lingait & Kumar [26]. The measurement procedure involved depositing 8 μL of deionized water droplet onto the film surface and capturing an image of the sessile drop.

#### 2.3.7. Water Vapor Permeability (WVP), Oxygen Permeability (OP), and Carbon Dioxide Permeability (CDP)

The method of Bizymis et al. [8] was used to determine the WVP of the film. A conical flask containing 20 mL of saturated sodium chloride solution (RH 75%) was sealed using the film (8 cm × 8 cm) and placed into a desiccator containing dried calcium chloride (RH 0%). The WVP was calculated using Equation (7).

(7) $$\mathrm{WVP}(g\cdot m^{-1}\cdot s^{-1}\cdot {\mathrm{Pa}}^{-1})=\frac{\Delta M\cdot d}{A\cdot t\cdot \Delta P}$$

where Δ*M* is the mass change over time t (g), *d* is the film thickness (m), *A* is the effective area for water vapor transmission (m^2^), *t* is the measurement time (s), and Δ*P* is the water vapor pressure difference across the sample (Pa).

The OP and CDP were determined using the method described by Wang et al. [28], with slight modifications. For OP determination, a conical flask containing 5 g of deoxidizer was sealed with the film sample and kept at 25 °C for 3 days to allow the measurement of weight change (ΔM) during the period. The deoxidizer was replaced with 20 mL of saturated potassium hydroxide (KOH) solution for CDP determination. The OP and CDP of the film were calculated using the following equations (Equations (8) and (9)):

(8) $$\mathrm{OP}(g\cdot m^{-1}\cdot s^{-1})\;=\;\frac{\Delta M\cdot d}{A\cdot t}$$

(9) $$\mathrm{CDP}((g\cdot m^{-1}\cdot s^{-1})=\frac{\Delta M\cdot d}{A\cdot t}$$

where Δ*M* is the weight increase (g), *d* is the film thickness (m), *A* is the effective area of the films (m^2^), and *t* is the testing time (s).

#### 2.3.8. Antioxidant Activity

Three pieces of film (25 mm × 25 mm) were immersed in 15 mL of deionized water and incubated at 25 °C for 24 h in a shaker incubator. The supernatant extract was used to determine the antioxidant activity by ABTS [2,2′-azino-bis(3-ethylbenzothiazoline-6-sulfonic acid)], DPPH (1,1-diphenyl-2-picryl-hydrazyl radical) and FRAP (ferric reducing antioxidant power) methods.

The ABTS free radical scavenging assay was performed according to the method of Monteiro et al. [27]. A solution containing ABTS (7 mM) and potassium persulfate (2.45 mM) was incubated in the dark at 25 °C for 16 h and diluted with deionized water to obtain an absorbance of 0.700–0.800 at 734 nm. The resulting solution (3.9 mL) was mixed with the film extract (0.1 mL) and incubated at 25 °C for 6 min. The absorbance was recorded at 734 nm using a spectrophotometer. The ABTS free radical scavenging activity was calculated using Equation (10).

The DPPH scavenging assay was conducted according to the method described by Yang et al. [29]. Briefly, 100 μL film extract was mixed with 3 mL of DPPH (0.1 mM methanol solution) and kept in darkness for 30 min. After incubation, the absorbance was measured at 516 nm, and the DPPH scavenging activity was calculated using Equation (10).

(10) $$\mathrm{Radical}\;\mathrm{scavenging}\;\mathrm{activity}\;(\%)=(1-\frac{A_{1}}{A_{0}})\times 100$$

where *A*_0_ is the absorbance of the ABTS or DPPH solution and *A*_1_ is the absorbance of the ABTS or DPPH solution with the film extract.

The FRAP of the film supernatant was determined according to the method described by Grzebieniarz et al. [30]. The FRAP reagent was freshly prepared by combining acetate buffer (pH 3.6), 20 mM ferric chloride, and 10 mM TPTZ (2,4,6-Tris(2-pyridyl)-1,3,5-triazine) (in 40 mM HCl) at a volume ratio of 10:1:1. For determination, 0.1 mL of the film extract was mixed with 3.9 mL of the freshly prepared FRAP reagent. The mixture was incubated in the dark at 37 °C for 30 min, and then the absorbance was measured at 595 nm. The results were calculated and expressed as mg of vitamin C equivalents per milliliter of film extract (mg Vc/mL).

#### 2.3.9. Fourier Transform Infrared Spectroscopy (FTIR)

The FTIR of the freeze-drying film was determined by following the method of Monteiro et al. [27]. FTIR spectra were recorded using KBr pellets by a Fourier infrared spectrometer (NICOLET 6700, Thermo Fisher Scientific, Waltham, MA, USA) within the wavenumber range of 500 cm ^−1^ to 4000 cm^−1^ at 4 cm^−1^ resolution and an average of 32 scans.

#### 2.3.10. Scanning Electron Microscopy (SEM)

The morphological and surface characteristics of the film samples were examined using a scanning electron microscope (ZEISS Sigma 300, Carl Zeiss AG, Oberkochen, Germany) following the method of Monteiro et al. [27]. Films were coated with gold using an SPI Module™ Sputter Coater (SPI Supplies, West Chester, PA, USA). The microstructures were observed and captured at an acceleration voltage of 5 kV for the surface and 3 kV for the cross-section. Micrographs were captured at magnifications of 1000× to assess overall surface homogeneity and finer microstructural details.

#### 2.3.11. X-Ray Diffraction (XRD)

The crystalline structure of the film was determined using an X-ray diffractometer (D8 ADVANCE, Bruker Corporation, Billerica, MA, USA). The XRD patterns were obtained with a scanning range of 2θ from 5° to 40° at a scanning speed of 1°/min.

#### 2.3.12. Release Kinetics of ε-Polylysine and Luteolin

To measure the ε-polylysine and luteolin released from the film, the film was cut into 6 cm × 6 cm squares and placed in 100 mL of 0.1 M citrate buffer (pH 3.5). At each designated time point (0 h, 4 h, 8 h, 20 h, 32 h, 48 h, 72 h, 96 h, and 144 h), 2 mL of the sample solution was taken and the remaining solution was replenished with buffer solution. The ε-polylysine content was determined using the methyl orange colorimetric method [30]. A ε-polylysine standard curve was used for the quantification. The luteolin content was determined by ultraviolet spectrophotometry at 354 nm and a standard curve was established using luteolin solutions of different concentrations.

### 2.4. Molecular Docking

AutoDockVina (version 1.2.3) was employed to simulate docking for the pectin–ε-polylysine, pectin–luteolin, ε-polylysine–luteolin, and pectin–ε-polylysine–luteolin complexes. A 20 mer unit of galacturonic acid (linked via 1,4-glycosidic bonds) was employed, with DE set at 50% (randomly carboxymethylating 10 galacturonic acid residues) for pectin, and ε-polylysine with a degree of polymerization of 15 (lysine residues linked via ε-bonds). Modeling was performed using ChemDraw (version 21.0.0) and Chem3D (version 21.0.0), with geometric optimization and energy minimization for docking. The structure of luteolin (CID: 5280445) was obtained from the PubChem Open Chemical Database (https://pubchem.ncbi.nlm.nih.gov/).

For pectin–ε-polylysine–luteolin docking, the receptor was the pectin–ε-polylysine complex (the optimal conformation after pectin–ε-polylysine docking). AutoDock Tools (version 1.5.7) were used for hydrogenation and polarity addition. The pectin–ε-polylysine complex with the lowest binding energy was selected. PyMOL (version 2.5.7) software was used to visualize the 3D docking results.

### 2.5. Application of Edible Coating on Fresh-Cut Apples

Apples of uniform size, which were free from mechanical damage or diseases, were washed, peeled, pitted and cut into cubes (2.0 cm × 2.0 cm × 2.0 cm). The cubes were immediately dipped into different coating solutions (Distilled water, NaCl solution, AP, APPL, and APPL-Lu (0.15%) for 15 s. After drying at room temperature, 20 cubes were put into a polypropylene box for one replicate and then stored at 4 °C and 75% RH conditions for 12 days. Each treatment contains 15 replicates. Samples were collected every 3 days to measure the quality index.

#### 2.5.1. Hardness

Fruit hardness was measured using the method described by Zhang et al. [31] with TA-XT plus texture analyzer. A P/5 probe with a diameter of 5 mm was used under the following operation conditions: a pre-test speed of 3 mm/s, a mid-test speed of 1 mm/s, a post-test speed of 3 mm/s, and a test distance of 3.5 mm, and the readings were measured in Newtons (N).

#### 2.5.2. Weight Loss

Using the weighing method [31], the weight loss rate (%) was calculated by Equation (11).

(11) $$\mathrm{Weight}\;\mathrm{Loss}\;\mathrm{rate}\;(\%)=\frac{W_{0}-W_{t}}{W_{0}}\times 100$$

where *W*_0_ is the weight of the apple on day 0 and *W_t_* is the weight of the apple on day *t*.

#### 2.5.3. Browning Index (BI)

The method of Said & Lee [32] was used to calculate the browning index. Color parameters (L*, a*, and b*) for each sample were measured using a colorimeter (CS-10, CHN Spec, Hangzhou, China). The BI was calculated using the following formula (Equations (12) and (13)):

(12) $$\mathrm{BI}\;(\%)\;=\;\frac{x-0.31}{0.172}\times 100$$

(13) $$\mathrm{BI}\;(\%)=\frac{x-0.31}{0.172}\times 100$$

#### 2.5.4. Total Phenols Content (TPC) and Total Flavonoids Content (TFC)

First, 1 g of apple pulp was added into 4 mL absolute methanol and the phenolic compounds were extracted by ultrasound at 40 °C for 30 min. The extract was centrifuged at 12,857*× g* at 4 °C for 15 min, and the supernatant was used for TPC and TFC analysis.

The Folin–Ciocalteu method was used to determine the TPC in the apple samples, as reported by Yang et al. [29], and the results were expressed as microgram of gallic acid equivalent per gram fruit (μg GAE/g).

The TFC was determined using the method described by Zhang et al. [33] with minor modifications. Briefly, 0.5 mL supernatant was mixed with 0.5 mL NaNO_2_ (5%) solution thoroughly. The mixture was kept at room temperature for 6 min, then 0.5 mL of Al(NO_3_)_3_ (10%) solution was added and the temperature was maintained for another 6 min. Afterward, 3 mL of NaOH solution (4%) was added and the solution was mixed well. The absorbance was measured at 510 nm after incubating for 20 min in darkness at room temperature. The results were expressed as microgram of rutin equivalent per gram fruit (μg RE/g).

#### 2.5.5. Total Soluble Solids (TSSs) and Titratable Acids (TA)

The TSS was measured with a refractometer at 20 °C. TA was determined by an acid–base titration method [31]. Briefly, 10 g of ground sample was homogenized with 50 mL of CO_2_-free water, boiled for 30 min, and filtered after cooling. A 25 mL aliquot of the filtrate was titrated with 0.1 M NaOH using phenolphthalein as the indicator, and the results were calculated as malic acid equivalent.

#### 2.5.6. Total Colony Count of Microbes from Apple Samples

The total plate count of microbes from apple samples was determined according to Lin et al. [34]. Briefly, 25 g of fruit pulp was homogenized in a specific volume of sterile saline. Ten-fold serial dilutions were prepared using sterile saline. Next, 0.1 mL dilution was spread onto Plate Count Agar plates, and the Colony-Forming Unit (CFU) was counted after incubation at 37 °C for 48 h.

### 2.6. Statistical Analysis

All experiments were conducted with at least three independent replicates (n ≥ 3). Data are presented as mean ± standard deviation. Statistical analysis was performed using R software (version 4.2.0). Prior to analysis, the assumption of homogeneity of variances was verified using the Levene’s test. One-way analysis of variance (ANOVA) was then employed to determine significant differences among treatment groups. When a significant F-value (*p* < 0.05) was obtained, post hoc multiple comparisons were carried out using the Waller–Duncan test. Differences were considered statistically significant at *p* < 0.05.

## 3. Results

### 3.1. Pectin–ε-Polylysine Films Incorporated with Different Phenolic Compounds

With the aim to screen effective phenolic compounds for functionalizing apple pectin–ε-polylysine-based edible coatings, the seven selected phenolic compounds were incorporated into the apple pectin–ε-polylysine (APPL) coatings, and the mechanical and antimicrobial properties of the composite films were evaluated.

#### 3.1.1. Mechanical Properties of Films Incorporated with Different Phenolic Compounds

**Figure 1:**
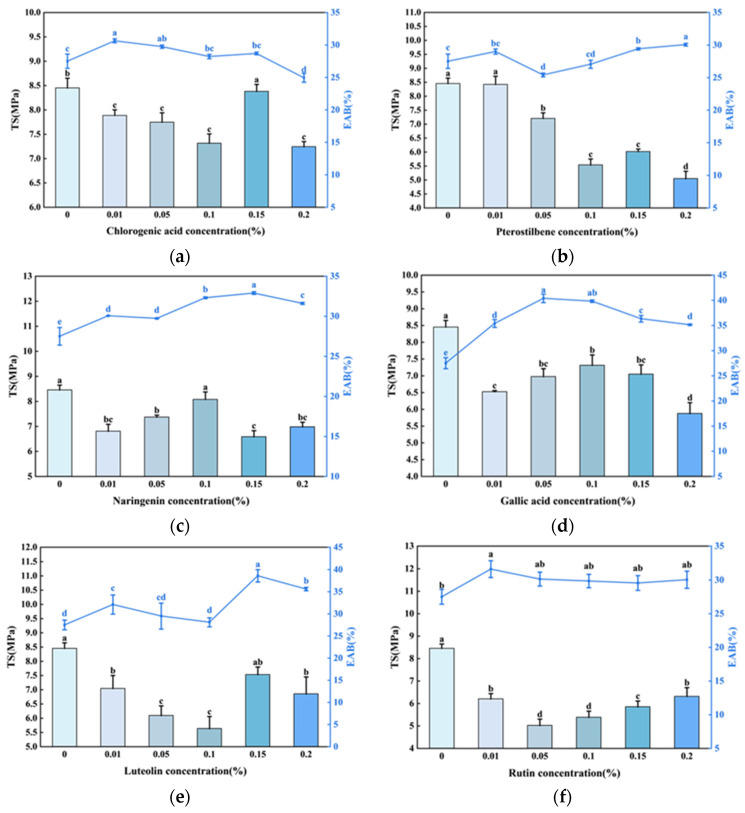

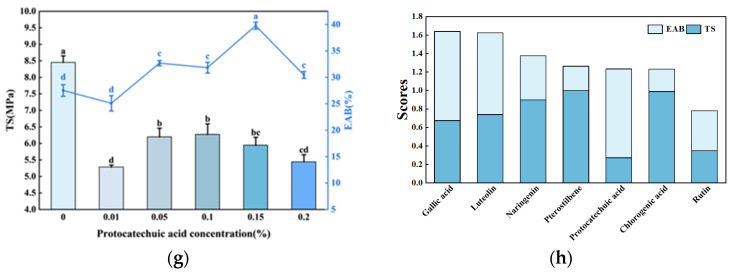
Mechanical properties of APPL films containing different phenolic compounds. (**a**) Chlorogenic acid; (**b**) pterostilbene; (**c**) naringenin; (**d**) gallic acid; (**e**) luteolin; (**f**) rutin; (**g**) protocatechuic acid; (**h**) scores of films containing different phenolic compounds at optimal concentration. TS: tensile strength (column legend); EAB: elongation at break (line legend). Different letters indicate a significant difference (*p* < 0.05).

The film formed by apple pectin (AP) alone had a tensile strength (TS) of 12.3 MPa and elongation at break (EAB) of 20.3%. Incorporation with ε-polylysine caused a significant decrease in TS and increase in EAB, reaching 8.45 MPa and 27.5%, respectively. The addition of phenolic compounds further altered the mechanical properties of the APPL films, with dependence on the phenolic compound as well as its concentration added in the film matrix. Different phenolic compounds showed diverse concentration-dependent patterns in terms of the TS and EAB of the functionalized films. As illustrated in Figure 1, the addition of phenolic compounds generally caused a decline in TS. Incorporation with gallic acid, protocatechuic acid, and naringenin showed similar trends of change in TS with the increase in concentration, increasing at lower concentrations (0.01–0.1%) and then declining from 0.1% to 0.2%. The highest TSs for these three phenolic compounds were all achieved at a concentration of 0.2% (Figure 1c,d,g). As for luteolin, chlorogenic acid, and pterostilbene, the TS decreased at concentrations of 0.01–0.1% and then showed an increase at a concentration of 0.15%, but the TSs tended to decline again from 0.15% to 0.2%. The optimal concentrations for these three phenolic compounds were 0.15%. The addition of rutin showed a declining tendency in TS at a concentration of 0.01–0.05% and then increased continuously from 0.05% to 0.2%.

Incorporation with the selected phenolic compounds generally improved the EAB of the films. As indicated in Figure 1, the EAB of protocatechuic acid and naringenin incorporated films generally showed an increasing tendency at concentration range of 0.01–0.15% and then declined from 0.15% to 0.2%. The gallic acid functionalized films exhibited an increasing trend at concentrations of 0.01–0.05% and then declined from 0.1% to 0.2%. The EAB of pterostilbene decreased at the concentration range of 0.01–0.05% and then increased from 0.05% to 0.2%. As for luteolin, the EAB showed a similar change trend to that of TS, and the highest EAB was achieved at a concentration of 0.15%. The EAB of chlorogenic acid-incorporated films declined slightly with the increase in concentration from 0.01% to 0.2%. No significant difference could be observed for rutin functionalized films among different concentrations.

To comprehensively evaluate the overall mechanical performance of different phenolic compounds that incorporated APPL, a Simple Additive Weighting (SAW) method [24] was employed to obtain a score for each treatment. As indicated in Figure 1h, gallic acid and luteolin that incorporated APPL at an optimal concentration achieved significantly higher scores than other samples, suggesting that they were more promising for improving the mechanical properties of APPL.

#### 3.1.2. Antimicrobial Properties

**Figure 2:**
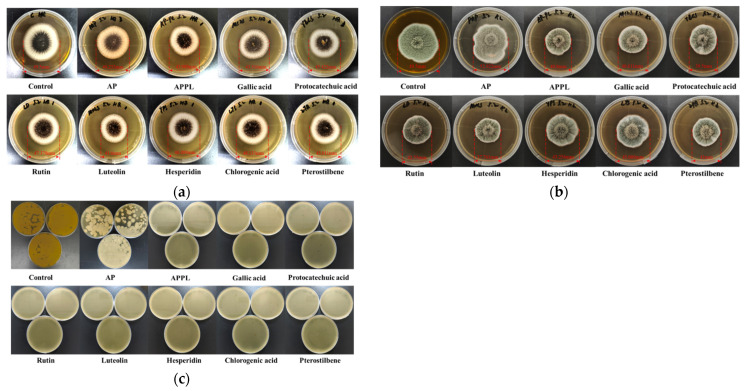
Effects of different coating solutions on the growth of (**a**) *Aspergillus niger*; (**b**) *Penicillium expansum*; (**c**) *Saccharomyces cerevisiae*.

As illustrated in Figure 2, the apple pectin (AP) solution exhibited little antimicrobial activity against *A*. *niger*, *P*. *expansum*,, and *S. cerevisiae*. After forming a complex with ε-polylysine and/or phenolic compounds, the coating solution showed significant inhibitory effects against these three microbial strains. The addition of luteolin yielded the strongest antimicrobial activity against *A*. *niger* and *P*. *expansum*, with inhibitory rates of 16.0% and 29.8%, respectively, which were significantly higher than those of the apple pectin–ε-polylysine (APPL) complex. This indicates that the incorporation of luteolin could significantly enhance the antifungal activity of APPL. Gallic acid also enhanced the antifungal activity against *A*. *niger* and *P*. *expansum*, but the inhibitory effects were much weaker than those of luteolin. On the other hand, both the APPL- and polyphenol-incorporated APPL treatments completely inhibited the yeast growth, suggesting that APPL has excellent anti-yeast activity in addition to its complexes with phenolic compounds. It should be noted that this research only compared the antimicrobial activity of different polyphenol-incorporated APPLs at the same level, without minimal inhibitory concentration (MIC) determination or comparative analysis against standard preservatives. This would affect the exact interpretation of the results. To better quantify and benchmark the antimicrobial efficacy, future studies should comprehensively evaluate antimicrobial activities by determining MIC and conducting comparative analyses against standard preservatives as well as using other methods.

### 3.2. Functionalized Pectin–ε-Polylysine Films with Luteolin

The above results suggest that luteolin- and gallic acid-incorporated APPL had better mechanical and antimicrobial properties. However, the antifungal activity of luteolin-APPL complex was much stronger than that of gallic acid, whereas their mechanical properties were comparable. Luteolin showed superior performance to gallic acid for functionalizing APPL film. Therefore, a functionalized pectin–ε-polylysine film with luteolin (APPL-Lu) was developed for the following experiments.

#### 3.2.1. Color and Transparency

Incorporation with luteolin significantly influences the optical properties of the pectin–ε-polylysine-based films. As listed in Table 1, there was no significant difference in the color and opacity parameters between AP and APPL. However, the lightness (L*) decreased significantly with the increase in incorporated luteolin concentration, while the redness (a*) and opacity significantly increased. The yellowness (b*) and ΔE increased at a lower luteolin concentration of 0.01–0.05%, but then tended to decline as the luteolin concentration increased further from 0.05% to 0.2%. These results suggest that the color of luteolin-incorporated pectin–ε-polylysine (APPL-Lu)-based films underwent a progressive shift in color to a yellowish-brown hue accompanied by a notable reduction in transparency with the increase in luteolin content.

#### 3.2.2. Mechanical Properties of Films Incorporated with Luteolin

Compared with APPL, the addition of luteolin resulted in a 10.89–33.37% decrease in TS depending on the luteolin content (Figure 1e). The highest TS was achieved following 0.15% addition of luteolin in the APPL. As for EAB, all the luteolin addition treatments generally resulted in an improvement, with 0.15% addition leading to the greatest improvement. The EAB of APPL incorporated with 0.15% luteolin was 38.6%, representing a 90.1% and 40.4% improvement as compared with AP and APPL.

This result is consistent with observations from pectin films plasticized with glycerol or gamma-aminobutyric acid (GABA), where the addition of these plasticizers also leads to decreased tensile strength (TS) and increased elongation at break (EAB) [35]. A plausible explanation is that the incorporation of ε-polylysine and luteolin modified the film’s microstructure by increasing the free volume and chain mobility of the polymer network, which in turn reduced its tensile strength. Therefore, improving the rigidity of the composite film will be a focus of our future work.

#### 3.2.3. Hydrophobicity

The hydrophobicity of APPL-Lu film was evaluated by determining the water absorption ability, water solubility, and water contact angle, with AP and APPL film as controls.

**Figure 3:**
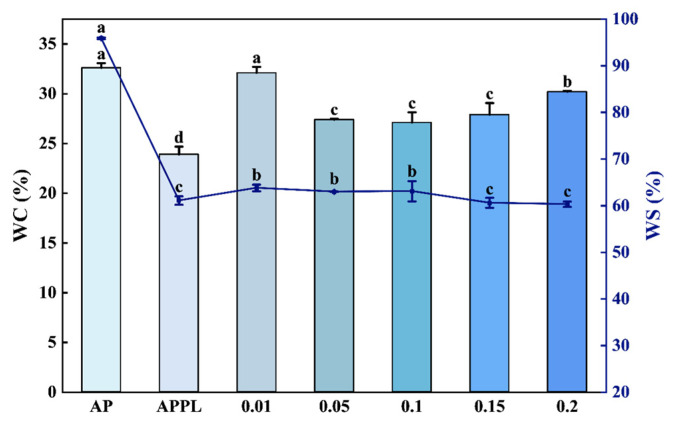
Water content (WC, in column legend) and water solubility (WS, in line legend) of different films. Different letters indicate a significant difference (*p* < 0.05).

The moisture content of films prepared with different apple pectin-based complexes after equilibrating at 50% RH for 48 h was shown in Figure 3. Higher moisture content indicates better water absorption ability. AP had the highest moisture content, and incorporation with ε-polylysine led to a significant decrease in moisture content, suggesting that a complex with ε-polylysine could reduce the water absorption ability of apple pectin-based film. Nevertheless, further incorporation with luteolin resulted in an increase in water absorption ability as compared with APPL, but the APPL incorporated with 0.05–0.2% luteolin was still lower than AP. The addition of luteolin might alter the state of water within the film, likely leading to a reduction in bound water associated with pectin and ε-polylysine and a corresponding increase in free water content [36]. The increased surface roughness of the film after luteolin addition also could result in a greater specific surface area, thereby increasing the number of sites available for water molecule adsorption and consequently elevating the film’s water content.

AP film had a high water solubility of 95% due to the hydrophilic nature of pectin. After a complex with ε-polylysine was formed, the water solubility was reduced to 61.1%. However, further incorporation with luteolin had little effect on the water solubility of the complexes. The lowest water solubility was observed at 0.15% and 0.2% addition of luteolin, which was decreased by 36.8% and 37.1%, respectively.

**Figure 4:**
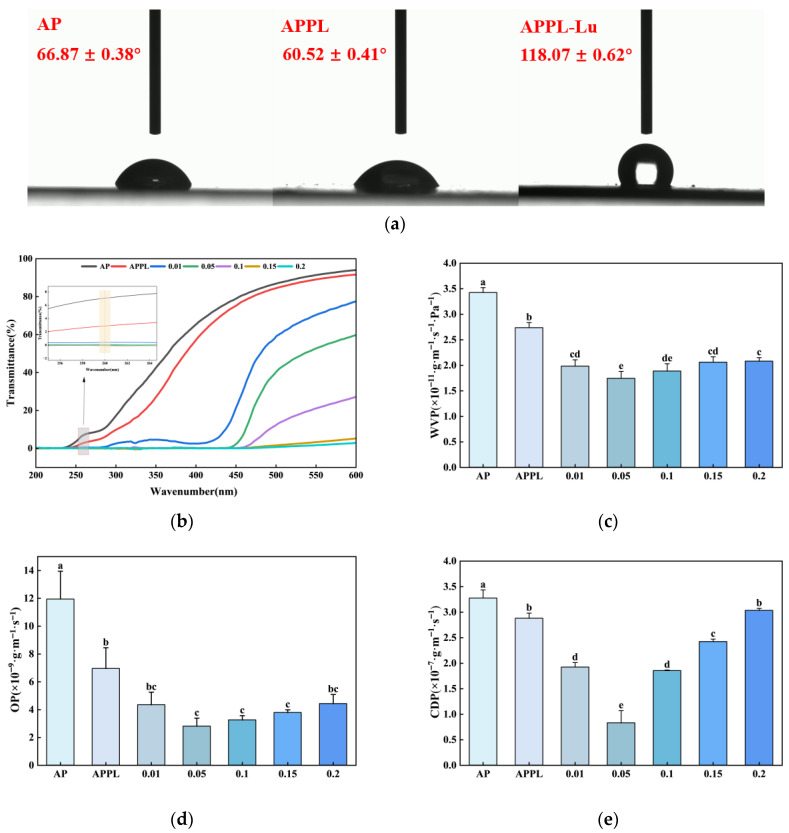
Water contact angles of the three film types and barrier properties of different films. (**a**) Water contact angles; (**b**) UV blocking performance; (**c**) WVP; (**d**) OP; (**e**) CDP. Different letters in each column indicate a significant difference (*p* < 0.05).

Water contact angle (WCA) measurements revealed significant changes in surface wettability following the incorporation of luteolin. As shown in Figure 4a, the AP and APPL had WCAs of 66.87° and 60.52°, indicating a hydrophilic characteristic. Upon the addition of luteolin, the WCA increased significantly to 118.07°, representing a notable enhancement in surface hydrophobicity. Surfaces with WCA greater than 90° represent high hydrophobicity and are classified as hydrophobic [37]. The incorporation of luteolin converted the film from hydrophilic to hydrophobic.

#### 3.2.4. Barrier Properties

The intensity of UV significantly affects food quality attributes and preservation potential. The optical properties of the films in the UV and visible regions were analyzed using a UV–visible spectrophotometer. As shown in Figure 4b, the AP and APPL films exhibited high optical transparency in the visible range. The addition of luteolin reduced the transmittance of the films in the 200 nm–600 nm wavelength range. The ultraviolet transmittance at 260 nm was 7.03% for AP and 2.89% for APPL. As the luteolin concentration increased, the transmittance gradually decreased to 0.22–0%. These results align with those of Bi et al. [38], demonstrating luteolin’s efficacy in enhancing film UV resistance. Their work provides mechanistic support, identifying the hydroxyl, phenyl, and carbonyl groups in luteolin as key contributors to UV absorption.

Water vapor permeability (WVP) is a critical parameter of films that directly affects food quality and shelf stability [39]. As shown in Figure 4c, the incorporation of ε-polylysine significantly reduced the WVP of the films. Furthermore, the WVP of the films exhibited a concentration-dependent pattern with the addition of luteolin, initially decreasing and then increasing. At luteolin concentration of 0.05%, the WVP reached a minimum value of 1.74 × 10^−11^ g·m^−1^·s^−1^·Pa^−1^, representing a 49.1% and 36.5% reduction as compared with AP and APPL film. However, at higher luteolin concentrations (0.1%, 0.15%, and 0.2%), the WVP of the film showed a slight increase, but the values were still much lower than those of AP and APPL films. At higher concentrations, the potential aggregation of luteolin could create voids within the biopolymer matrix, thereby adversely affecting its WVP [40]. The WVP of the APPL-Lu film developed in this study was significantly lower than that of the pectin film incorporated with mulberry leaf extract (5.59 ± 0.012 g·m^−1^·s^−1^·Pa^−1^) reported by Shivangi et al. [23]. This notable improvement highlights the exceptional barrier properties achieved by this novel ternary composite coating, positioning it as a highly competitive candidate for high-performance food preservation.

Oxygen permeability (OP) and carbon dioxide permeability (CDP) are essential parameters for evaluating the barrier performance of edible films and coatings. Lower OP and an appropriately matched CDP can help to regulate the respiration rate of fresh produce, thereby extending its shelf life [37,41]. As shown in Figure 4d,e, the incorporation of both ε-polylysine and luteolin effectively reduced OP and CDP of the composite films. This observed reduction in OP and CDP followed a trend consistent with WVP behavior, suggesting a coordinated enhancement of the film’s barrier properties against different gas molecules. At luteolin concentration of 0.15%, the composite film exhibited a significant reduction in CDP, showing decreases of 26.5% and 10.7% compared to the AP and APPL groups, respectively. OP was decreased by 68.6% and 42.8% as compared with AP and APPL, respectively. This enhancement in barrier performance indicates that the incorporation of luteolin contributed to the formation of a more compact film structure, thereby improving its overall gas barrier properties. Compared to the PLA/pectin bilayer film (OP = 2.2 × 10^−7^ g·m^−1^·s^−1^) reported by Said et al. [32], the APPL-Lu film demonstrates superior oxygen barrier properties.

#### 3.2.5. Antioxidant Property

**Figure 5:**
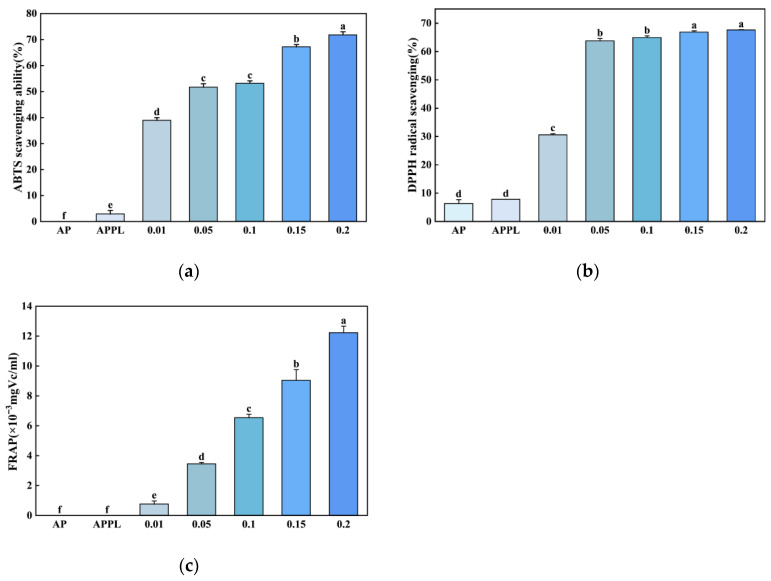
Antioxidant properties of different films. (**a**) ABTS; (**b**) DPPH; (**c**) FRAP. Different letters in each column indicate a significant difference (*p* < 0.05).

The antioxidant activity of the film was examined via three in vitro methods. With the exception of the ABTS assay, no significant differences in antioxidant capacity were observed between APPL and AP (Figure 5). The incorporation of ε-polylysine did not confer inherent antioxidant activity to the pectin matrix. Additionally, the antioxidant activity of the film increased with the rising luteolin dosage across all three systems, indicating that luteolin supplementation conferred strong antioxidant properties to the film. It has been reported that luteolin and its derivatives exhibit potent antioxidant activity [42]. The DPPH and ABTS scavenging activities, as well as the FRAP values of the film leachate, exhibited obvious concentration-dependent relationships with luteolin concentration. This is consistent with the findings of Sutharsan et al. [43], who reported an increasing trend of antioxidant activity of chitosan films with the elevation of flavonoids concentrations incorporated in the films.

#### 3.2.6. Simulated Release

**Figure 6:**
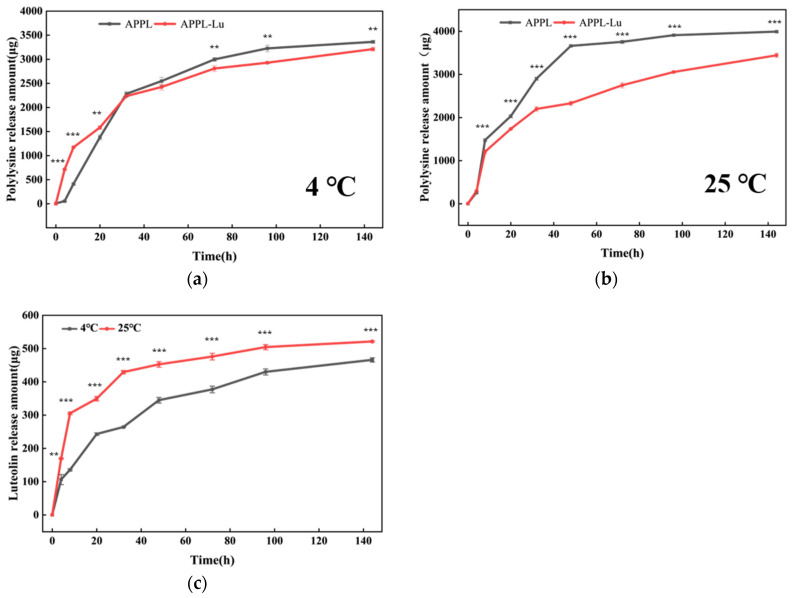
Release kinetics of ε-polylysine and luteolin at 4 °C and 25 °C. (**a**) ε-polylysine at 4 °C; (**b**) ε-polylysine at 25 °C; (**c**) luteolin at 4 °C and 25 °C. Statistical significance between the two groups was determined by Student’s *t*-test: ** *p* < 0.01, *** *p* < 0.001.

Simulated release not only serves as an indirect indicator of the underlying structure of the APPL and APPL-Lu composites but also holds significant importance for their further practical applications. Therefore, the release rate of ε-polylysine from both APPL and APPL-Lu into the environmental solution was determined. Considering that the pH range of apple juice typically falls between 3.0 and 4.0 [44], the experiments were performed under simulated conditions of pH = 3.5. As shown in Figure 6a,b, ε-polylysine release was observed in both APPL and APPL-Lu (0.15%). After 144 h, the ε-polylysine release rates were 1.94% in APPL and 1.86% in APPL-Lu at 4 °C, whereas they were increased to 2.31% in APPL and 1.99% in APPL-Lu at 25 °C, respectively. Compared to 4 °C, the films exhibited faster ε-polylysine release rates at 25 °C and APPL-Lu exhibited a slower release rate.

The release kinetics of luteolin at 4 °C and 25 °C are shown in Figure 6c. A rapid release was observed within the first 20 h, with cumulative amounts reaching 242.8 μg (1.50%) and 349.3 μg (2.2%), respectively, in 100 mL of simulated solution. The luteolin release amounts at 144 h were 465.9 μg (2.9%) and 521.3 μg (3.2%), respectively. The slower release rate in the later stage enables the system to provide sustained release of luteolin. The significantly slower release rate observed at 4 °C compared to 25 °C aligned with the pattern seen for ε-polylysine. This temperature dependence is likely due to increased molecular diffusivity and enhanced polymer chain mobility at higher temperatures, which facilitate the diffusion of active compounds [45].

Due to the prolonged and controlled release of active compounds, the film ensures persistent antioxidant and antimicrobial efficacy during the post-harvest storage of fruits and vegetables, resulting in a significant extension of shelf life [46].

#### 3.2.7. Structural Characterization

**Figure 7:**
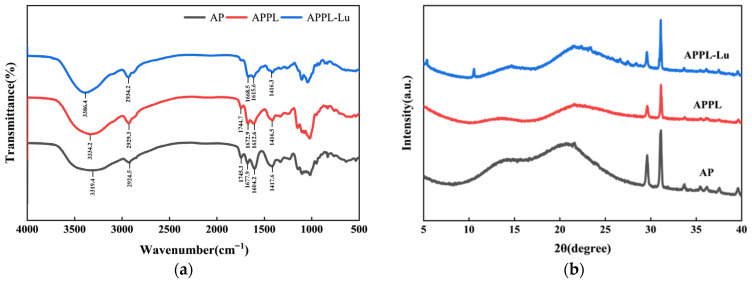

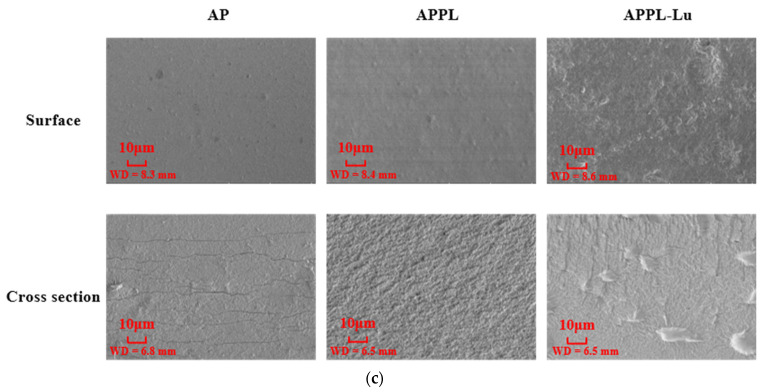
Structural characterization of the films by (**a**) FTIR, (**b**) XRD, and (**c**) SEM.

The structure of the AP, APPL, and APPL-Lu films was characterized by FTIR, XRD, and SEM. The results are presented in Figure 7.

The FTIR analysis reveals a consistent pattern of molecular interactions across different spectral regions, elucidating the formation mechanism of the composite films. In the 3200–3500 cm^−1^ region, associated with O–H and N–H stretching vibrations [47], both APPL and APPL-Lu exhibited distinct red shifts relative to AP, moving from 3386.4 cm^−1^ to 3334.2 cm^−1^ and 3319.4 cm^−1^, respectively (Figure 7a). These shifts indicate the formation of hydrogen bonds involving the amide groups of ε-polylysine and hydroxyl groups from both pectin and luteolin. Complementary evidence emerges in the lower-frequency regions. In the carbonyl stretching region (~1745 cm^−1^), APPL showed a minor redshift (1745.3 cm^−1^ to 1744.7 cm^−1^), which was consistent with electrostatic interactions between ε-polylysine and pectin. Notably, this peak was obscured in APPL-Lu, suggesting strong hydrogen bonding between luteolin’s phenolic hydroxyls and pectin’s ester carbonyl groups [48]. The carboxylate region (~1600 cm^−1^) provides direct evidence of progressive network strengthening. The AP film showed a characteristic peak at 1604 cm^−1^, which could be attributed to relatively free carboxylate vibrations. Upon ε-polylysine incorporation (APPL), this peak blue-shifted to 1612 cm^−1^, demonstrating restricted carboxylate vibrations due to electrostatic interactions [49,50]. Further incorporation of luteolin (APPL-Lu) induced an additional blue shift to 1615 cm^−1^, confirming reinforcement of the network through extensive hydrogen bonding without disrupting the pre-existing ionic cross-links. Collectively, the consecutive blue shift from 1604 cm^−1^ to 1615 cm^−1^, coupled with the observed red shifts in hydroxyl and carbonyl regions, systematically demonstrates the cooperative reinforcement of the film network through both electrostatic interactions and hydrogen bonding.

Figure 7c showed the SEM results of the films. Compared to AP, APPL exhibits a smoother surface. On the other hand, a significant alteration in surface morphology was observed for the APPL-Lu (0.15%) film, which showed a noticeably rougher texture compared to the control. This phenomenon could be attributed to the addition of luteolin, which migrated to the surface and underwent localized aggregation during film formation, resulting in the creation of micro-scale protrusions [51]. These surface features increased roughness and, consequently, enhanced hydrophobicity, a conclusion well-supported by the higher water contact angle measurements.

Cross-sectional observation revealed that all three film formulations exhibited a certain degree of void formation. However, upon the incorporation of ε-polylysine and luteolin, a notable reduction in both the size and number of these pores was observed. APPL-Lu demonstrated the most compact microstructure, characterized by the fewest and smallest pores among all samples (Figure 7c). This may be because the small-molecule luteolin, through appropriate arrangement and intermolecular forces, fills the composite network formed by pectin and PL, thereby imparting dense and continuous network integrity to the composite film [52].

XRD analysis revealed a broad, flat peak around 20° for all three films (Figure 7b), indicating that APPL composite film did not form a regular crystalline structure. The modification of the pectin film by ε-polylysine lies not in the creation of a new crystalline structure, but in the establishment of a more robust and dense ionically cross-linked network within the amorphous pectin matrix. This reinforced network is primarily responsible for the enhanced mechanical and barrier properties. Analysis of the spectra revealed that luteolin, originally a highly crystalline pure substance, transformed into an amorphous state within the composite film. This indicates strong molecular interactions between luteolin and APPL matrix, rather than simple physical mixing [53]. The molecular chains were arranged in a disordered manner, and after adding luteolin, they did not form an independent crystalline structure. The entire system maintained an amorphous structure. These results confirm significant molecular-level interactions between luteolin and APPL matrix, and indicate the molecular-level dispersion of luteolin. This alteration might partly account for the increased elongation at break of the composite film.

#### 3.2.8. Molecular Docking Results

**Figure 8:**
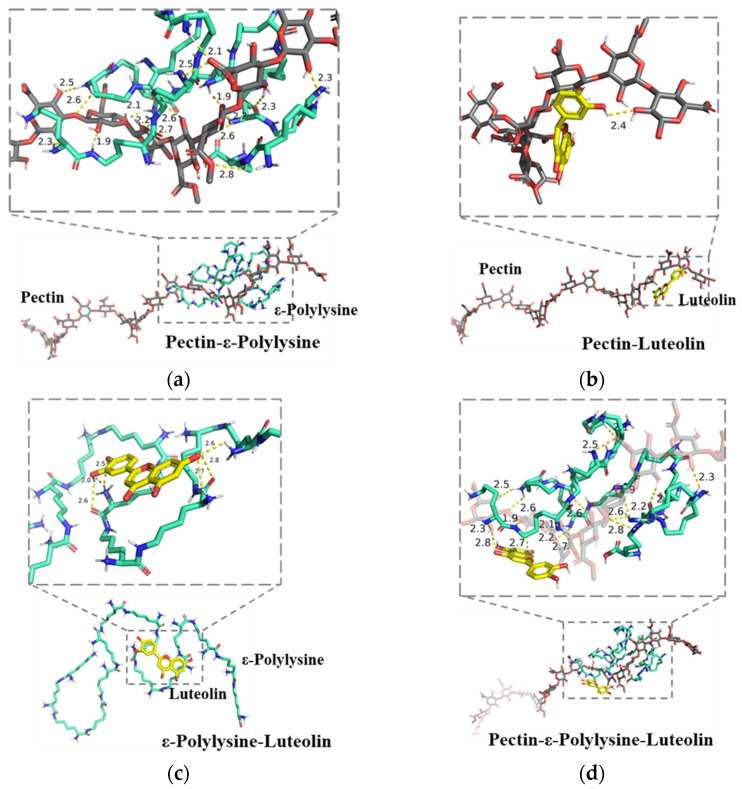
Molecular docking results. (**a**) Pectin with ε-polylysine; (**b**) pectin with luteolin; (**c**) ε-polylysine with luteolin; (**d**) luteolin with the pectin–ε-polylysine complex.

To elucidate the potential molecular interactions among pectin, ε-polylysine, and luteolin, molecular docking simulation was performed, and the best docking results were presented in Figure 8.

ε-polylysine was found to spontaneously bind to pectin with a predicted binding energy of −3.6 kcal/mol. Visualization analysis revealed that the binding is primarily driven by electrostatic interactions, particularly the formation of multiple salt bridges between the negatively charged carboxylate groups of galacturonic acid residues on pectin and the protonated amino groups in the ε-polylysine side chains. In addition, an extensive hydrogen-bond network was observed between the two macromolecules. ε-polylysine adopted an extended conformation along the pectin chain, and this binding mode showed good consistency across multiple docking runs. These results computationally demonstrate a strong direct interaction between the two biopolymers, also providing a molecular-level explanation for the sustained release of ε-polylysine.

As shown in Figure 8b,c, luteolin also exhibits a significant binding affinity to both pectin and ε-polylysine, with binding energies of −4.7 kcal/mol and −4.5 kcal/mol, respectively. Notably, its binding energy with the pre-formed pectin–ε-polylysine complex was −5.5 kcal/mol (Figure 8d). The incorporation of luteolin thus further stabilizes the composite system, offering a molecular-scale rationale for the superior mechanical and barrier properties observed in the ternary composite film.

### 3.3. Preservation of Fresh-Cut Apples with Pectin–ε-Polylysine–Luteolin Coating

To assess the applicability of the apple pectin–ε-polylysine–luteolin (APPL-Lu) complex for preservation of fresh produce, experiments on fresh-cut apples coated with AP, APPL, and APPL-Lu were conducted, and the effects on fruit quality attributes, including browning index, hardness, weight loss, total soluble solids (TSSs), titratable acids (TAs), total colony count, total phenols, and flavonoids, were determined.

#### 3.3.1. Browning Index

**Figure 9:**
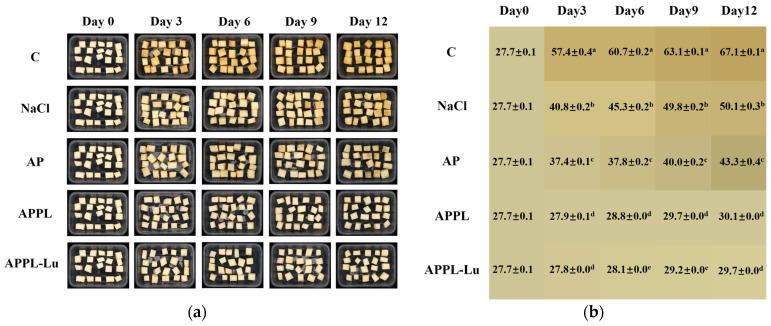
Appearance (**a**) and browning index (**b**) of fresh-cut apple cubes with different treatments during cold storage at 4 °C. Different letters in each column indicate a significant difference (*p* < 0.05).

Browning induced by both enzymatic and non-enzymatic reactions is a primary issue in the preservation of fresh-cut apples [54]. As shown in Figure 9, noticeable browning was observed in the control, NaCl treatment, and AP groups during 12 days of cold storage at 4 °C, with a final browning index of 67.1%, 50.1%, and 43.3%, respectively, representing increases of 142.2%, 80.9%, and 56.3% as compared with the initial value. NaCl treatment and AP coating retarded the browning process to some extent. The APPL and APPL-Lu treatment groups showed little change in color appearance, with a final browning index of 30.1% and 29.7%, respectively, which was only 8.66% and 7.22% higher than the initial value. These markedly lower values confirm that the incorporation of ε-polylysine and luteolin significantly enhanced the anti-browning activity of the pectin-based film.

#### 3.3.2. Hardness of Fresh-Cut Apples

**Figure 10:**
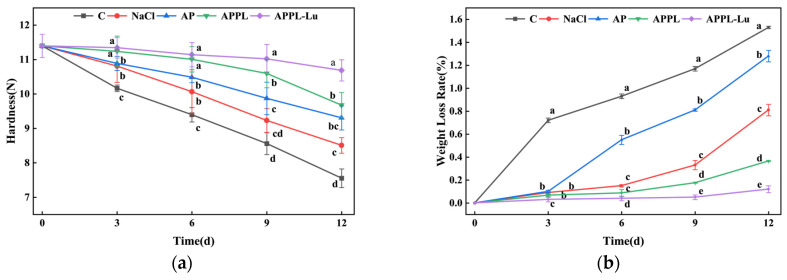

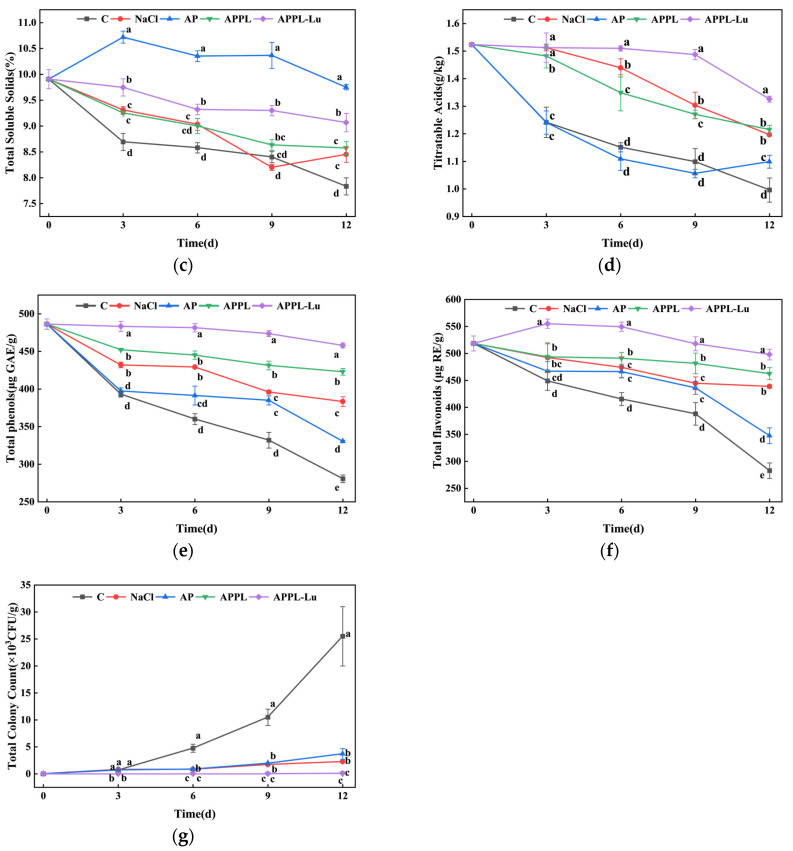
Quality changes of fresh-cut apples with different treatments during cold storage at 4 °C. (**a**) Hardness; (**b**) weight loss; (**c**) total soluble solids; (**d**) titratable acids; (**e**) total phenols; (**f**) total flavonoids; (**g**) total colony count. Different letters in each column indicate a significant difference (*p* < 0.05).

The hardness of fresh-cut apples declined during cold storage. As illustrated in Figure 10a, the control and NaCl treatment groups showed a quick softening change during storage, with hardness decreasing by 33.3% and 25.4% at the end of storage compared with the initial hardness. The hardness of the AP treatment group also decreased continuously during the process with a lower speed than the control and NaCl treatment groups, suggesting that AP coating treatment could suppress the softening of fresh-cut apples during storage. Coating with APPL and APPL-Lu further inhibited the softening of fresh-cut apples, resulting in higher hardness than other groups. The final hardness of the APPL and APPL-Lu groups was 9.7 N and 10.7 N, respectively, which was only 15.1% and 6.2% lower than the initial hardness. On the other hand, the hardness was 28.1% and 41.5% higher than the control, suggesting that the APPL and APPL-Lu coating could better retain the hardness of fresh-cut apples.

#### 3.3.3. Weight Loss of Fresh-Cut Apples

The fresh-cut apples showed continuous weight loss during storage, primarily due to moisture evaporation and respiratory consumption of dry matter [55]. As shown in Figure 10b, the weight loss rate of fresh-cut apples in all groups continuously increased during cold storage. The control groups showed more rapid weight losses than others, yielding a weight loss rate of 1.5% at the end. AP, APPL, and APPL-Lu coating treatments retarded the weight loss speed, resulting in a weight loss rate of 1.2%, 1.3%, and 0.12%, respectively, at the end of storage. These results suggest that coating with AP, APPL, and APPL-Lu could effectively prevent the weight loss of fresh-cut apples during cold storage. In particular, regarding the APPL-Lu coating treatment, the weight loss could be reduced to an unnoticeable level.

#### 3.3.4. Total Soluble Solids and Titratable Acids

Total soluble solids (TSSs) and titratable acids (TAs) were two essential indicators of the nutritional and flavor properties of fresh fruits. As illustrated in Figure 10c,d, the TSS and TA of the fresh-cut apples generally showed a decreasing trend across all apple groups during storage except for the AP group, which showed an increase in TSS in the initial storage period (0–3 d). This might be attributed to the high water solubility of AP, which would allow more low-molecular-weight pectin fractions present in the coating solution to be likely measured as part of the soluble solids content. The incorporation with ε-polylysine and luteolin decreased the solubility of AP, thereby mitigating the influence of coating solutions on TSS determination. The significantly slower TSS and TA decline in APPL-Lu groups compared to that in other groups might be ascribed to the low OP and CDP of the coatings, which would suppress the respiration rate and metabolic activity during storage.

#### 3.3.5. Total Phenols and Flavonoids

The total phenols and flavonoids showed similar descending trends during cold storage, probably due to the oxidative degradation of phenolic compounds (Figure 10e,f). The fruits with the APPL-Lu coating exhibited the slowest decreasing speed both in total phenolic and flavonoid contents, resulting in significant higher levels than those observed for other groups. The APPL coating treatment also slowed down the decreasing speed of total phenolic and flavonoid contents, but was less effective than APPL-Lu. AP coating treatment even had a limited preventive effect on phenolic degradation, only yielding a 17.7% higher total phenols content and 22.9% higher total flavonoid content than the control groups. Besides the barrier effect to oxygen transmission, the better retention of total phenols and flavonoids in apples coated with APPL-Lu may be partly attributed to the introduction of luteolin, which elevated the phenolic and flavonoid content in the fruit and effectively inhibited their loss as an antioxidant. Similar results were also reported by Zha et al. [56], who found that riboflavin inhibits browning in fresh-cut apples by suppressing phenolic metabolism and enhancing the antioxidant system to mitigate peroxidation, thereby preserving quality.

#### 3.3.6. Total Colony Count

Microorganisms causing food spoilage are a primary reason for the deterioration of fresh-cut apples [57]. As shown in Figure 10g, both the APPL and APPL-Lu groups significantly reduced the total colony count in the stored fresh-cut apples, showing excellent antibacterial properties. Numerous studies have demonstrated that active coating treatments could dramatically inhibit microbial growth on fruits [58,59,60]. These results clearly demonstrate that the inherent antimicrobial property of ε-polylysine effectively endowed the pectin-based film with antibacterial functionality. At the same time, the incorporation of luteolin provided a distinct synergistic enhancement to the film’s overall efficacy. Subsequent research will focus on dedicated studies against key foodborne pathogens and dominant spoilage fungi to explicitly assess the coating’s safety implications and delineate its specific antimicrobial spectrum.

## 4. Discussion

Pectin is generally recognized as safe (GRAS) by the FDA (Food and Drug Administration, USA) and can be employed as film-forming material for elaborating edible coatings and films to prolong the shelf life of fresh produce [61]. Owing to its high hydrophilicity and water solubility as well as limited bioactivity, pectin is usually combined with other polymers or active ingredients such as chitosan, protein, antimicrobials, and antioxidants to form composite films and coatings with enhanced performance [9]. The present research demonstrates that incorporation with the selected phenolic compounds improved the elongation while decrease the tensile strength, which is in agreement with previous research [62]. Specially, luteolin incorporated pectin–ε-polylysine film exhibited excellent performance in terms of both enhancement of mechanical strength and antimicrobial activity. The addition of 0.15% luteolin increased the EAB of the pectin film from 20.3 ± 0.331% to 38.6 ± 1.39% which was significantly higher than the value of 32.15 ± 2.74% reported for the pectin film incorporated with 15% GABA [35]. This indicates that the composite coating possesses superior flexibility, damage tolerance, and adhesion to the irregular and delicate surface of fresh-cut fruits. This enhanced toughness ensures the coating to maintain its integrity during handling, storage, and consumption, which is critical for sustained preservation efficacy, rather than merely exhibiting high strength under ideal conditions.

The incorporation of luteolin also enhanced the hydrophobicity, as evidenced by the increase in WCA from 66.87 ± 0.38° to 118.07 ± 0.62°. Compared to conventional pectin-based films which typically exhibit WCA below 100° [63], the APPL-Lu film demonstrates exceptional hydrophobicity. This superior water-repellent property enables it to effectively lock the moisture within fresh-cut fruits and resist external water, thereby contributing significantly to the preservation quality. Compared to the single-pectin film, the enhanced barrier performance and antioxidant capacity of the composite film enable it to more effectively regulate gas exchange and inhibit water vapor diffusion, thereby suppressing the respiration and metabolic activities of the coated fruits. This mechanism is highly favorable for preserving fruit quality.

Mechanistic investigations revealed the interactions among pectin, ε-polylysine, and luteolin within the ternary system, which could be responsible for the superior characteristics of the functionalized composite coating. Electrostatic interactions between pectin and ε-polylysine serve as the structural framework. FTIR analysis revealed a significant blue shift in the ~1600 cm^−1^ region, from 1604 cm^−1^ to 1612 cm^−1^, which is attributed to the strong electrostatic attraction between the carboxylate groups of pectin and the protonated amino groups of ε-polylysine [64]. This finding is further corroborated by molecular docking results, which revealed multiple electrostatic interaction sites between pectin and ε-polylysine. Such electrostatic cross-linking forms the structural basis of a dense three-dimensional network, leading to the initial improvement in mechanical and barrier properties. The reinforcement of the hydrogen bonding network further stabilizes the structure. After complex of ε-polylysine with pectin, a blue shift was observed in the O–H/N–H region (~3319 cm^−1^ to ~3334 cm^−1^) due to hydrogen bond electrostatic interactions formation between the two components [65]. Upon the incorporation of luteolin, this peak underwent a further blue shift from 3334 cm^−1^ to 3386 cm^−1^, accompanied by an additional blue shift in the carboxylate region from 1612 cm^−1^ to 1615 cm^−1^. The hydroxyl groups of luteolin act as additional cross-linking points to form extensive hydrogen bonds with the carboxyl/hydroxyl groups of pectin and the amide/amino groups of ε-polylysine, thereby reinforcing the electrostatic complex between pectin and ε-polylysine [66]. Molecular docking results showed an increase in the binding energy among the three components after the introduction of luteolin, indicating that the additional hydrogen bonds strengthen the polymer network, which accounts for the remarkable optimization of the film’s mechanical and barrier properties. An absence of distinct crystalline peaks characterizes a glassy or amorphous material, suggesting lack of long-range ordered crystal structure [67]. The XRD pattern of the APPL film showed minor shifts compared to that of AP, indicating good compatibility between ε-polylysine and the pectin matrix through intermolecular interactions [65]. The XRD pattern of luteolin exhibited sharp crystalline peaks at 2θ values of 5°, 10°, 22.4°, 23.5°, 26.2°, and 29.8° [68]. The transformation of luteolin from a crystalline to an amorphous state upon its incorporation into the pectin–ε-polylysine matrix suggests strong interactions between luteolin and the matrix [69]. These intermolecular interactions contributed to a more compact film structure. SEM images (Figure 7c) revealed that the APPL-Lu film possessed smaller and fewer pores. This denser film structure consequently resulted in superior gas barrier properties. The surface of the luteolin-added film appeared rougher than that of its counterpart, likely due to varying degrees of luteolin aggregation on the surface, which also led to the exposure of hydrophobic groups and, consequently, a significant increase in film hydrophobicity.

The developed pectin–ε-polylysine–luteolin ternary composite coating demonstrated robust fresh-keeping efficacy, primarily achieved through synergistic effects among its components. ε-polylysine incorporation provided the pectin-based matrix with strong antibacterial capacity, enabling the ternary coating to effectively suppress mold and yeast growth. Complementing this, luteolin contributed excellent antioxidant activity to the system while simultaneously inhibiting polyphenol oxidase [70], leading to significant browning reduction. The slow, sustained release of both ε-polylysine and luteolin was integral to the coating’s long-term efficacy. Coated fresh-cut apples maintained a desirable color and appearance throughout the 12-day storage period. Moreover, the ternary composite coating treatment further preserved the nutritional quality of the apple cubes through mechanisms including respiration retardation [71] and metabolic modulation [72].

However, several limitations must be acknowledged. Scaling up production may pose challenges related to raw material standardization, reaction consistency, and cost control. The light yellowish hue of the film could also affect consumer acceptance when applying to light-colored foods. Only seven phenolic compounds were screened in this research, which just could provide limited knowledge on the interaction of phenolic compounds with pectin or pectin–ε-polylysine matrix. Future studies should focus on scaling protocols, conducting life-cycle assessments, and investigating the film’s applicability to a wider range of food matrices. Additionally, consumer studies and detailed cost–benefit analyses will be crucial for industrial translation, and screening more phenolic compounds may help to find more effective ones for functionalizing pectin-based coatings.

## 5. Conclusions

In this study, we successfully created a ternary composite coating by incorporating ε-polylysine and luteolin into a pectin matrix. The functionalized coating exhibited enhanced barrier properties, accompanied by significantly improved mechanical strength and hydrophobicity. It also exhibited remarkable functional activities, including potent antimicrobial efficacy and exceptional antioxidant capacity.

The application of the ternary composite coating on fresh-cut apples demonstrated that it successfully inhibited browning and microbial growth while preserving hardness and nutritional quality. Compared to controls, the treated samples exhibited enhanced soluble solids content, total phenol and flavonoid retention, decreased weight loss, and improved acidity stability, demonstrating excellent quality preservation through multifunctional actions.

Mechanistic investigations revealed that these characteristics are due to the molecular interactions within the ternary system. The FTIR, XRD, and SEM analyses combined with molecular docking simulations confirmed the formation of synergistic electrostatic complexes and hydrogen-bonding networks. These interactions promoted the development of a densely organized matrix structure with uniform component distribution, enabling simultaneous improvement of barrier properties and sustained release profiles.

Therefore, the ternary composite coating represents a promising and multifunctional alternative to conventional preservation materials. To translate this promising proof-of-concept into practical application, future investigations into industrial-scale production, cost-effectiveness, and consumer acceptance will be essential.

## Figures and Tables

**Table 1:** Color and transparency of APPL films incorporated with different concentrations of luteolin.

Parameter	AP	APPL	APPL Incorporated with Different Concentration of Luteolin (%)				
			0.01	0.05	0.10	0.15	0.2
L*	84.2 ± 1.38 ^a^	82.4 ± 2.40 ^b^	74.2 ± 2.24 ^c^	69.7 ± 1.85 ^d^	63.4 ± 2.67 ^e^	60.3 ± 2.86 ^f^	57.0 ± 3.78 ^g^
a*	1.32 ± 0.384 ^f^	1.25 ± 0.439 ^f^	5.47 ± 1.40 ^e^	8.79 ± 1.28 ^d^	12.3 ± 1.70 ^c^	13.8 ± 1.50 ^b^	16.5 ± 2.59 ^a^
b*	17.0 ± 3.08 ^f^	18.3 ± 2.83 ^f^	50.8 ± 3.10 ^e^	59.2 ± 1.21 ^d^	54.0 ± 2.63 ^c^	46.4 ± 3.37 ^b^	39.0 ± 7.38 ^a^
ΔE	17.4 ± 3.33 ^d^	19.3 ± 3.62 ^d^	52.7 ± 3.73 ^c^	62.6 ± 0.851 ^a^	61.1 ± 0.913 ^a^	56.7 ± 0.883 ^b^	53.9 ± 7.07 ^c^
Opacity	0.284 ± 0.068 ^e^	0.340 ± 0.0460 ^e^	0.788 ± 0.128 ^e^	1.97 ± 0.273 ^d^	4.55 ± 0.753 ^c^	9.63 ± 1.19 ^b^	11.4 ± 1.35 ^a^
Image	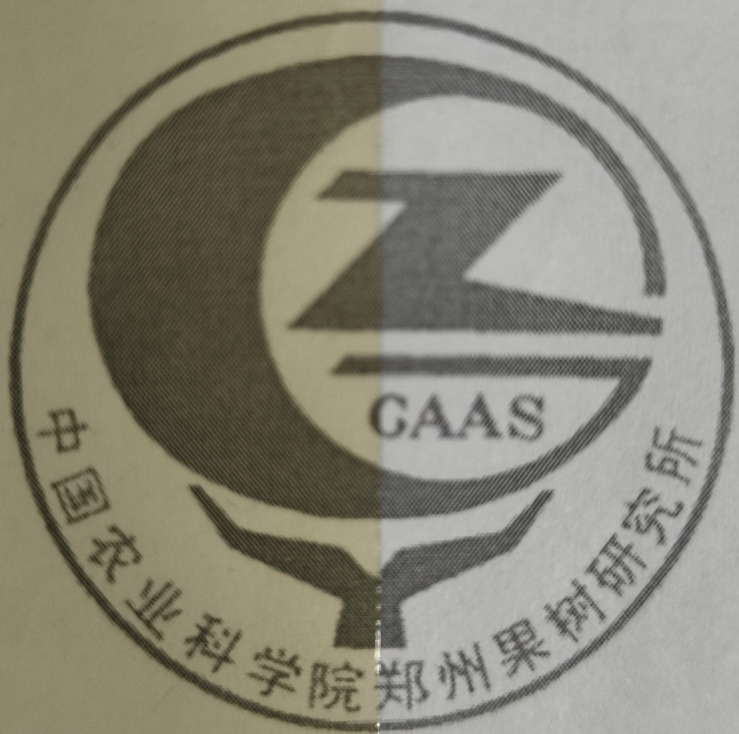	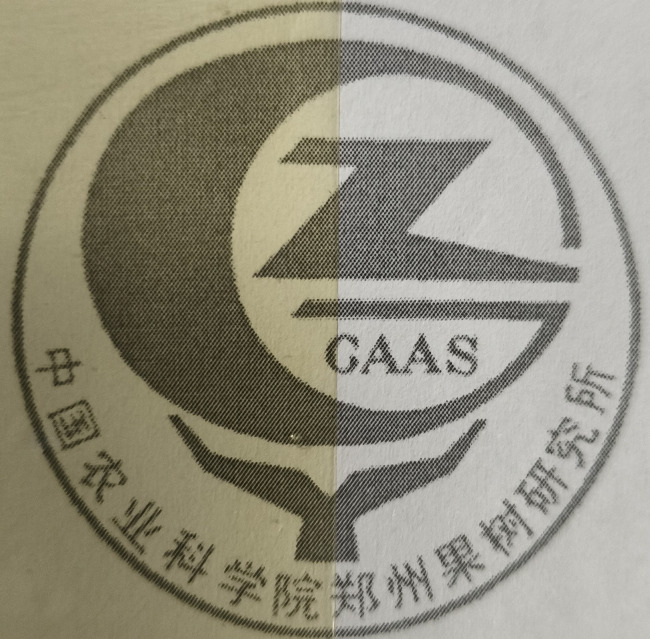	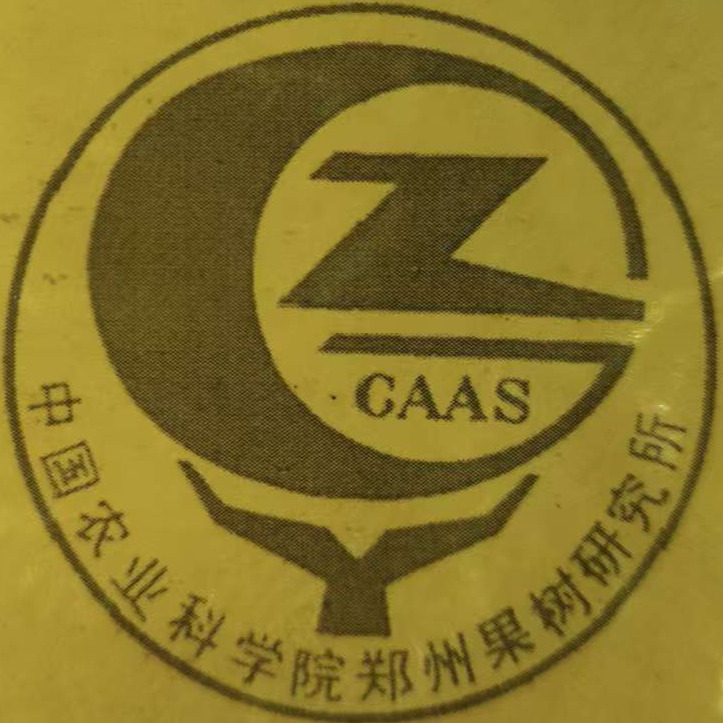	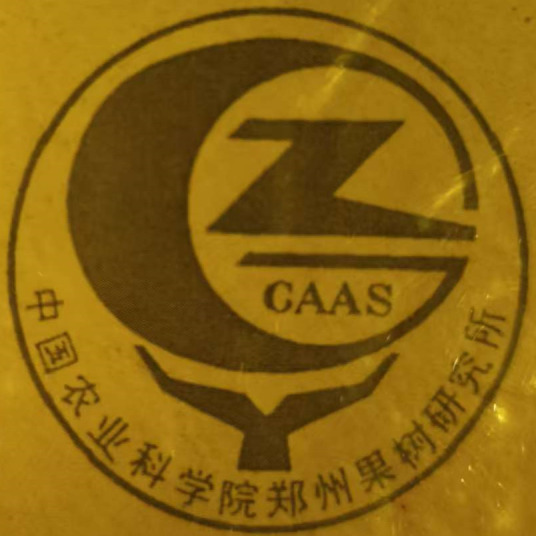	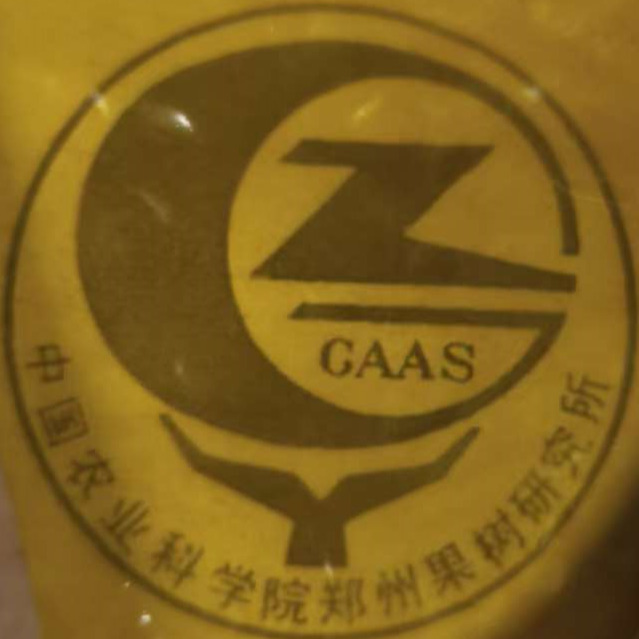	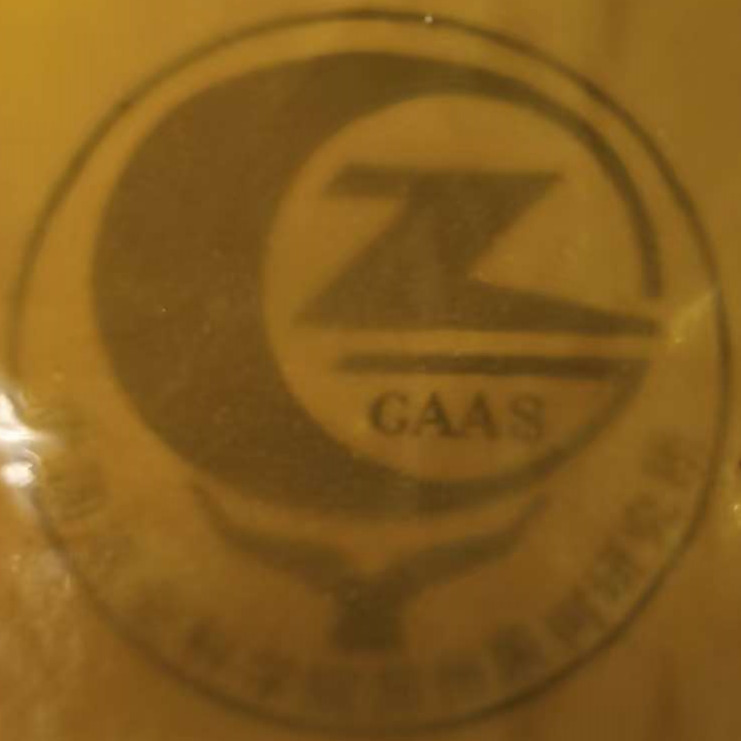	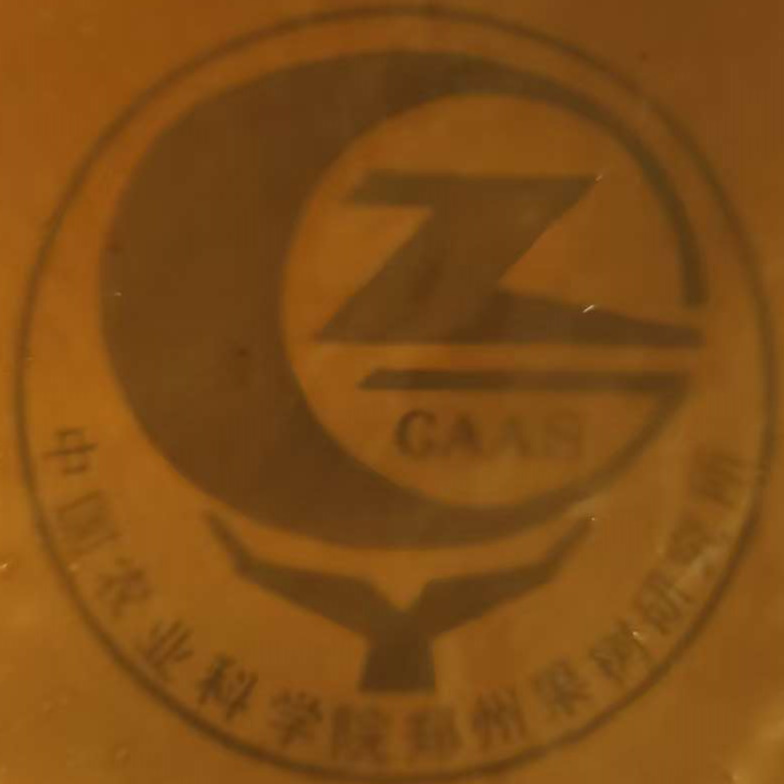

Different superscript letters in the same line indicate a significant difference (*p* < 0.05).

## Data Availability

The original contributions presented in this study are included in the article. Further inquiries can be directed to the corresponding author.

## Abbreviations

The following abbreviations are used in this manuscript:

AP	Apple pectin.
APPL	Apple pectin–ε-polylysine complex.
APPL-Lu	Apple pectin–ε-polylysine–luteolin complex.
TS	Tensile strength.
EAB	Elongation at break.
WCA	Water contact angle.
WVP	Water vapor permeability.
OP	Oxygen permeability.
CDP	Carbon dioxide permeability.
FTIR	Fourier transform infrared spectroscopy.
SEM	Scanning electron microscopy.
XRD	X-ray diffraction
